# Postpartum onset Takayasu’s arteritis presenting with aortic dissection

**DOI:** 10.1093/omcr/omae078

**Published:** 2024-07-30

**Authors:** Hirotaka Yamamoto, Yoshinori Taniguchi, Yujiro Miura, Shigeto Kobayashi

**Affiliations:** Department of Endocrinology, Metabolism, Nephrology and Rheumatology, Kochi Medical School Hospital, Kochi University, 185-1 Kohasu, Oko-cho, Nankoku, Kochi 783-8505, Japan; Department of Endocrinology, Metabolism, Nephrology and Rheumatology, Kochi Medical School Hospital, Kochi University, 185-1 Kohasu, Oko-cho, Nankoku, Kochi 783-8505, Japan; Department of Cardiovascular Surgery, Kochi Medical School Hospital, Kochi University, 185-1 Kohasu, Oko-cho, Nankoku, Kochi 783-8505, Japan; Department of Internal Medicine (Rheumatology), Juntendo Koshigaya Hospital, 560 Fukuroyama, Koshigaya, Saitama 343-0032, Japan

**Keywords:** rheumatology

## Abstract

Takayasu’s arteritis (TA), also known as pulseless disease and young female arteritis, is a chronic inflammatory large-vessel vasculitis (LVV). TA is pathologically characterized by arterial wall thickening, stenotic/occlusive lesions, aneurysm formation, and dissection. TA usually develops between 20 and 30 years of age. However, pregnancy and puerperium can affect the immune system, and several cases of postpartum onset or flare-up of TA have been reported. Herein, we report an extremely rare case of postpartum-onset TA complicated by aortic dissection. This is a case of Postpartum onset Takayasu’s arteritis presenting with aortic dissection. A 34-year-old healthy woman was performed cesarean section. After 2 weeks, she presented with chest pain and fever, followed by mild dysphagia and hoarseness. Laboratory findings showed C-reactive protein (CRP) 21.61 mg/dl and computed tomography (CT) demonstrated thickening of the vessel wall of mainly ascending aorta. 18F-fluorodeoxyglucose (FDG)-position emission tomography (PET)/CT revealed high FDG uptake in the same areas. We diagnosed with TA and steroid pulse therapy was started. However, five days after treatment, the patient developed worsening symptoms of hoarseness. A contrast-enhanced CT showed Stanford A type dissection, and emergency artificial vessel replacement was performed. The specimen from surgical resection of the ascending aorta suggested active TA associated with dissection. The prednisolone dosage was gradually tapered with tocilizumab. Then, her symptoms and laboratory findings improved. It is important to recall the onset of TA and/or arterial dissection, when patients develop chest pain and hoarseness in the postpartum period.

## Introduction

Takayasu’s arteritis (TA), also known as pulseless disease and young female arteritis, is a chronic inflammatory large-vessel vasculitis (LVV). TA is pathologically characterised by arterial wall thickening, stenotic/occlusive lesions, aneurysm formation, and dissection [1]. TA usually develops between 20 and 30 years of age. However, pregnancy and puerperium can affect the immune system, and several cases of postpartum onset or flare-up of TA have been reported [2, 3]. Herein, we report an extremely rare case of postpartum-onset TA complicated by aortic dissection.

## Case report

A 34-year-old healthy woman with no clinical symptoms, including fever or blood pressure changes during pregnancy, underwent caesarean section at full term. Two weeks later, she presented with chest pain and fever, followed by mild dysphagia and hoarseness. Physical examination revealed that blood pressure was 124/88 mmHg with no difference between right and left sides, pulse 104/min, SpO_2_ 96% (2 L oxygen), and body temperature 38°C. She had left neck tenderness, a Levine 1 degree total systolic murmur at the apex of the heart, wheezing during expiration, and pitting oedema of the lower limbs. Urinalysis showed no proteinuria and hematuria. Laboratory findings noted high C-reactive protein (CRP, 21.61 mg/dl), white blood cell count 38 300/μl and erythrocyte sedimentation rate (ESR, 140 mm/h) with normal levels of hepatic and renal biological markers. Serum complement levels were elevated. The test results for antinuclear antibody, myeloperoxidase-antineutrophil cytoplasmic antibody, proteinase 3-antineutrophil cytoplasmic antibody, and rheumatoid factor were negative. The T-SPOT®.TB test and blood culture tests were negative. Electrocardiogram showed normal sinus rhythm without ischemic signs. Echocardiography demonstrated normal left ventricular (LV) size and normal LV systolic function.

**Figure 1:**
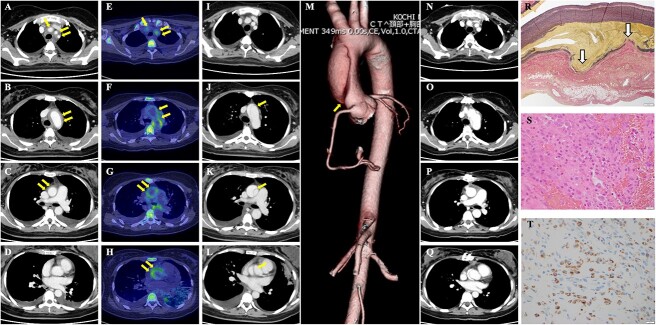
Imaging and pathological findings of TA with aortic dissection. CT images indicating thickening of the vessel wall (arrows) from the ascending aorta to the right common carotid artery, left internal carotid artery, and left subclavian artery (**A**–**D**). FDG-PET/CT scan revealing high FDG uptake (**E**–**H**, arrows) in the areas exhibiting abnormal findings on CT. Contrast-enhanced CT images demonstrating Stanford A type dissection from the ascending aorta to aortic arch (**I**–**M**, arrows). CT images performed artificial vessel replacement of the dissection site (**N**–**Q**). Biopsy specimen from the surgical resection of the ascending aorta indicating dissection of the tunica media close to the outer membrane side (Elastica van Gieson staining) (**R**, arrows) and inflammatory cells infiltration (**S**). Immunostaining revealing infiltration of CD68 positive histiocytes (**T**). TA, Takayasu’s arteritis; FDG, 18F-fluorodeoxyglucose.

Computed tomography (CT) revealed thickening of the vessel wall from the ascending aorta to the right common carotid, left internal carotid, and left subclavian artery (Fig. 1A–D, arrow). Further, 18F-fluorodeoxyglucose (FDG)-PET/CT revealed high FDG uptake in the same areas (Fig. 1E–H, arrow).

Based on these findings, we diagnosed the patient with postpartum-onset TA. Intravenous infusion of methylprednisolone (1 g daily) was started for three days, followed by oral prednisolone (PSL) 80 mg (1 mg/kg) daily. However, five days after treatment, the patient developed worsening symptoms of hoarseness and dysphagia. Repeat contrast-enhanced CT showed dissection from the ascending aorta to the aortic arch, indicating the Stanford A type (Fig. 1I–M, arrow), and emergency artificial vessel replacement was performed (Fig. 1N–Q). The specimen from the surgical resection of the ascending aorta demonstrated dissection of the tunica media close to the outer membrane side (Fig. 1R, arrow) and inflammatory cell infiltration, mainly CD68 positive histiocytes (Fig. 1S and T) on the outer membrane side. These pathological findings suggest that an active TA is associated with dissection. The postoperative course was uneventful, and the PSL dosage was gradually tapered. A subcutaneous injection of tocilizumab (162 mg weekly) was initiated for TA as an additional treatment. Her symptoms and laboratory and imaging findings improved completely without flares.

## Discussion

In the present case, the condition was first-onset postpartum TA with dissection. First, postpartum-onset TA is rare, and some cases have been reported [2, 3]. Why did postpartum onset TA develop? Although the mechanism of pathogenesis is unclear, postpartum hormone changes and the release of immune tolerance may be triggering factors. For example, high estrogen levels during pregnancy promote a pro-inflammatory T helper (Th) 1/Th17 response shift toward a Th2/regulatory T cell response and suppress the production of tumor necrosis factor, interleukin (IL)-1, and IL-6 [4]. In addition, progesterone suppresses the function [5], both of which play important roles in immune tolerance during pregnancy. During the postpartum period, the release of these immune tolerances can exacerbate Th1-dominant diseases, such as TA, multiple sclerosis, and rheumatoid arthritis. In the present case, in addition to the human leukocyte antigen (HLA)-B52 genetic factor, postpartum release of immune tolerance and hormonal changes may have initiated the onset of TA.

Second, why does postpartum TA develop into a dissection? Recently, the clinical significance of pregnancy-related aortic dissection has been noted. Postpartum onset is especially noticeable in the perinatal period and is associated with a high mortality risk [6]. It has been hypothesized that estrogen and elastase alter the structure of the vascular wall during pregnancy, causing the rupture of reticular fibers, changes in the arrangement of elastic fibers, and proliferation and hyperplasia of smooth muscle cells in the aortic tunica media, resulting in a friable aortic wall [7]. TA is also an important factor in pregnancy-related aortic dissection [8], which is caused by necrosis of smooth muscle cells, destruction or loss of elastic fibers in the tunica media, worsening of vascular fragility, and increased blood pressure due to reduced elasticity in the outer membrane fibrosis [9].

Finally, what are the risk factors of aortic dissection in patients with TA? In childhood TA, the risk factors could be a higher pain visual analogue score, increased neutrophil count, lower albumin levels, and arterial hypertension [10]. Other studies in giant cell arteritis as LVV-like TA indicated that symptoms of aortitis (dorsal/lumbar/abdominal pain, aortic insufficiency) and high disease activity could be a factor in aortic complications [11]. Similarly, TA before the dissection development showed high disease activity based on serological and imaging findings in the present case. Thus, in the present case, the potential vascular fragility due to hormonal changes during pregnancy was further exacerbated by the hyperactive TA caused by the release of postpartum immune tolerance, resulting in aortic dissection. Thus, not only increased disease activity of TA but also the changes in vascular structure are more likely to develop during the postpartum period, and therefore might result in possible dissection.

In conclusion, this case demonstrates that it is important to recall the onset of TA and/or arterial dissection, when patients develop postpartum chest pain and hoarseness.

## Ethical approval

Patient information has been de-identified and consent for publication has been obtained.

## Consent

This case report has been published with the written consent.
